# On the Relative Worth of Symptoms, with Some Remarks on Borax

**Published:** 1889-09

**Authors:** C. Von Bœnninghausen


### ON THE RELATIVE WORTH OF SYMPTOMS—WITH SOME REMARKS ON BORAX.

(Dr. C. Von Bœnninghausen, translated by Carroll Dunham, M. D., from the *Allgemeine Homœopathische Zeitung*, vol. 53, 1856.)

Among the various elaborations of the *Materia Medica Pura* of Hahnemann of which such an abundance have been made in modern times, I miss one whose importance has only of late years become fully evident to me. This is a statement of the *time* which elapsed *after the taking of the remedy before a given symptom was observed.* Passing over the value or worthlessness of all other alleged deficiencies—although younger critics have put forth nothing better or more serviceable—I have in view to say something on this topic only, because it appears to me to be of no little importance in practice.

If my old (seventy-two years) memory does not mislead me, it was the genial C. Hering—I do not recall when or where—who first (and up to the present time he is the only one who has called my attention to it) pointed out to me that the *proving-symptoms* which manifested themselves *last* were the *most important* for *employment in curing,* and were far from being only secondary and useless in therapeutics.

Certainly, at the first glance, there seems to be a paradox in this, as in many other things, that this indefatigable investigator has asserted. But to be willing to form an apodictic opinion prematurely, from the mere aspect of the thing, would in this case be all the more unreasonable, because every homœopath can, without great difficulty, obtain in the records of the provings sufficient certainty of the correctness or falsity of this assertion. He needs only to compare, in the four volumes (second edition) of *Antipsoric Remedies,* certain symptoms which were latest observed, with the brief indications which were given by Hahnemann himself in his introduction to each proving from his individual experience as pre-eminently belonging to these remedies, and which have been abundantly verified as such in our practice. He will thereby be convinced that the analogue to these indications is in most cases contained, and sometimes exclusively, in such *late-observed* symptoms.

A truth appears, therefore, to lie at the bottom of this assertion of Hering's, which till now has been little observed, and which makes us regret that, in so many new as well as old provings, so little attention has been paid to a statement of the *time* at which the symptoms manifested themselves after the taking of the drugs, and especially in the case of those peculiar symptoms in which mainly the individual characteristics of the drug must be sought. Although the fact that a knowledge of the importance of such a statement of the time must have been reserved to later comparative studies may serve as an excuse for former provers, yet this omission is not on that account less worthy of regret, and we are often obliged to first discover by the long process of experience that which might then have been supplied us by the putting together of some little figures and letters.

It may be interesting to consider the above-mentioned observations with regard to other remedies also—namely, to such as are seldom used, and about which Hahnemann has left us no especial teachings in this respect. Among others, *Borax* appears particularly fitted for such investigation (*Chron. Krankheit* II, 28), where the time of the phenomena is almost throughout sufficiently specified in the symptoms observed by Dr. Schreter. I think I may be allowed, therefore, as a proof of the above general remarks, to make a few observations upon it which may serve at the same time as a contribution to the more precise characteristics of this perhaps too-much-neglected remedy. If therein I deviate from the newer (so-called scientific) fashion of proper treatment of the subject, I beg my readers to remember that my object here is only an especial and limited one, and above all that I make no secret of belonging to the old Hahnemannian school (now almost extinct).

BORAX.

At the very beginning in both symptoms 4 and 5, of which the first was observed during five weeks, the second during three weeks, a peculiarity meets us which belongs to no other remedy in the same way. It is *anxiety* on *sudden downward motion,* and is by no means to be confounded with the but slightly similar symptoms which we have of Carb. veg., Sep., and Sulph. According to my experience, this anxiety clearly expresses itself in a *swing,* and most pre-eminently at the moment when the swing moves *forwards,* almost never when it moves *backwards.* I have observed this by no means unusual symptom not only in children, but also in two adult women, and in every case regarded it as a useful one, and it also by the result proved itself to be of value not simply for this, but also for the other existing trouble.* Sickness from riding, especially on

* I should too far overstep the limits of this communication if in every case I should particularize the form of disease. I therefore confine myself to briefly stating that one of these patients, a woman of thirty years, suffered from difficult menstruation; the other, a strong woman of forty, from frequent attacks of erysipelas.

the back seats, as well as sea-sickness, has little in common with this, and evidently Borax will not be of use in these cases, although in some forms of the latter this remedy might well be tried.

2. Not less characteristic appears symptom 7 (without statement of time) as regards *violent fright from the report of a gun, even at a distance*, and I mention this only as it were in passing, because in my experience it is an excellent remedy for *hunting-dogs* which are *shot-shy*, a fault which, as my hunt-loving colleagues know, occurs not seldom, and is often difficult to cure. Moreover, there are persons, especially children, who start at every shot and receive from it a great and unnatural fright. Just so, excessive fear of *thunder* appears to belong here.

3. Among the symptoms which affect the eye, we come upon two, viz.: 77 and 78, which are pre-eminently peculiar to this remedy, and which until now were observed only in the working of *Silic.* and *Puls.* It is that peculiar kind of inflammation of the eyes which is caused and kept up by the *growing-in of the eye-lashes,* thus constantly irritating the ball of the eye, and which is not permanently cured even when allopathically the corpus delicti has been removed, and the lashes torn out by the roots. Every one of us has probably found the admirable working of Borax proved in many cases of this kind of inflammation (of course, the other symptoms must correspond), and it only remains to be noticed that symptom 77 was first observed *after six weeks* and symptom 78 *after thirty-five days.*

4. Among the morbid phenomena of the ears from symptom 88 to 106, and in connection with which symptoms 51 and 60 must be considered, *those* have, by the curative results, proved themselves to be the most marked which were connected with *ulceration of the ear.* But these are symptoms 95, 96, 97, which first showed themselves *on the twenty-seventh day* and *on the nineteenth day.* Symptom 51, just mentioned in this connection, first appeared after the *thirty-second day,* and at the same time with symptom 96.

5. Scabs in the nasal cavities, with inflammation and shining redness at the tip of the nose, which are not seldom met with in (psoric) persons who have neither at any time been syphilitic nor been abused with *Merc.*, often find (with *Sep.* or *Sil.*) their remedy in Borax, as many, also, of us may have experienced. But the symptoms which apply here—109, 111, and 112—do not stand among those which appear in the first days after taking the remedy, but date from the *tenth, sixteenth, and eighteenth days.* It is probable that many among us have, like myself, had opportunity to cure by means of this remedy painful *erysipelas*, commonly on the left side of the face (the similar Bell. erysipelas generally occupies the *whole* face or only the *right half*) which is unendurable, especially when the muscles contract in laughter. The two symptoms which apply here—120 and 121—were not observed until from the *thirty-first* to the *thirty-fourth* day.

7. Of the toothaches which are cured quickly and lastingly by Borax, I recall only the one which corresponds with symptoms 137 and 139, connected with symptom 133 by reason of the influence of chilly weather, and with symptom 136 on account of aggravation by cold water. I call attention to the fact that the two first-named symptoms appear on the *fortieth day*.

Moreover, this remedy, according to symptoms 147 and 148, and in connection with symptom 125, is not unfrequently very successful in the *teething of children*, in which cases it must be ranked among the most useful of our remedies; especially in cases in which the symptoms 150 to 153* are present at the same time. Here also I remark that the symptoms 147 and 148 were observed after *forty* and *thirty-six* days respectively.

8. Borax has long been known to allopathy as a frequently efficient remedy in aphthous mouth-affections of children, the practice being to pencil the mouth with it. Every one of *us*, too, have seen its satisfactory operation in this often very troublesome disease of childhood, when, in other respects, it has been homœopathically chosen—that is, when there has been no contra-indication. There can consequently be no doubt of its relative curative action in this connection. Now, the four symptoms which relate to this affection in the proving all appeared very late, viz.: 150 *after four weeks;* 121 *after thirty days;* 152 *after thirty-three days*, and 153 *after five weeks.*

9. Symptoms 218 to 223 correspond with great distinctness to a form of spleen-affection, and, indeed, with clear and apparently exact indications which would appear to insure the correct choice of the remedy in any given case. I must confess, nevertheless, that I have never seen in any kind of spleen-affection any result worth mentioning from the administration of Borax; and I mention this fact here only for this reason, because the symptoms to which I refer were all observed *very early*, and, indeed, *only a few days after the drug was taken*, and only symptom 22 occurred after fifteen days. This negative fact seems worthy of some notice.

---

* Aphthæ on the inside of cheeks, which bleed during a meal, and on the tongue.

10. Among the *urinary* symptoms—267 to 280, together with 434—those have best and oftenest approved themselves in practice, to *me*, at least, which were latest observed. Among these belong especially the *frequent urination at night*, observed, according to 268, *after twenty-four days*, and according to 434, *after thirty-four days*. The same is true of the symptoms occurring *after* micturition as detailed in 275 to 280—among which the *soreness in the urethra* has presented itself to me as the most constant. Symptom 276 gives this as occurring *after the thirtieth day*, and 278 *after the twenty-sixth day*.

11. According to my experience the preference is to be given to Borax in *too early* and *too long-continued menstruation;* although with this remedy, as with many others, the tardy appearance or short continuance of the discharge is no contra-indication. The first-mentioned peculiarity, however, is represented by symptom 294, as observed *after twenty-five days*, and by 295, *after seven weeks*.

12. Among the chest symptoms, the most marked is a painful affection of the intercostals, especially of the right side, with which the cough and respiratory symptoms stand in immediate relation—as well as the sneezing (34) and the sleep symptoms (435). Although the majority of these symptoms occurred within the *first eight days* after taking the drug, it must yet be observed that the question here concerns almost exclusively an *acute* affection, and that, nevertheless, symptom 349, according to which an aggravation *when lying on the* (*right*) *painful side* occurs, had lasted *full four weeks*. The contradictory symptom 435, which states the contrary, but which experience shows to have but little value, and which has never been corroborated in my experience, was observed *within seven days*. I think myself warranted in saying, therefore, that Borax can be profitably administered only in recent acute attacks of this nature, and in such I have never tried it, since for these other approved remedies stand us in stead.

13. Although Galactorrhœa of nursing women occurs under several drugs (Acon., Bell., Bry., Calc., Chin., Con., Iod., Lyc., Phos., Puls., and Rhus), I have, nevertheless, in repeated cases found symptom 360, *which occurred on the thirty-second day*, of approved value, especially where, in addition to other corresponding symptoms, there was present the "unpleasant feeling of emptiness in the emptied (sucked-out) breast," which is mentioned in 360, and which occurs under no other remedy.

14. We have possessed hitherto, so far as I know, only one remedy which corresponds to the ulcers on the backs of the finger and toe-joints, such as not unfrequently occur in chronic (psoric) patients, viz.: Sepia; for under Nux vomica relief in such cases is to be expected only in the finger-joints, and is seldom permanent. Borax furnishes us a second and a very efficient remedy, according to symptoms 385, the time of which is not given, 387 observed *after thirty days*, and 405 *after fifteen days*. It may be remarked here that, other symptoms corresponding, Borax deserves the preference when, according to symptom 408, the skin is very *unhealthy*, and the ulcer corrodes in its periphery, which is not wont to be the case in these ulcers under Sepia, at least to the same extent. Especially for children is Borax suitable.

15. It may *finally* be observed that the predominant *chilliness* which is quite peculiar to this drug, and furnishes an excellent indication for its use, was likewise observed *very late*, viz.: *after twenty-three, fourteen, thirty-three days;* and even *after five weeks.*

To avoid confounding the *uncertain* with the *proved*, I have confined myself in the foregoing statements to a comparatively small number of established facts, although surely among the effects of Borax are to be found many other objects of cure. What has been said, however, is quite sufficient to accomplish my immediate object, and to show that the statement of Hering, referred to in the beginning, is based upon *fact*, and is strikingly confirmed by experience. There is, therefore, abundant reason for a caution against the practice of regarding, as many do, the *late-observed symptoms*, especially of long-acting drugs, as *secondary* or *curative* symptoms; an overhastiness, of which, though for the most part he subsequently corrected it, Hahnemann was guilty in some of his earlier provings, without at that time suspecting his error, and in consequence of this some symptoms still remain distinguished by this *mala nota*.

On pain of being charged with heresy by our younger colleagues, who, disregarding the repeated and urgent warnings of Hahnemann, operate only with low potencies and with frequent doses, I, by reason of my many-yeared and wide experience, hesitate not to affirm distinctly and assuredly that precisely those morbid phenomena which are *deepest-rooted* are most quickly, most surely, and most permanently cured by the administration of such remedies as (if appropriate in other respects) furnish corresponding indications among their *latest observed symptoms;* and especially is this the case when these remedies are given in *very high* potencies, and in *small and infrequent doses*. Whoever has experienced the contrary, let him communicate his experience unreservedly but truthfully—for only by the honorable and frank interchange of manifold, even be they contradictory, experiences, can we bring the entire simple *truth* into the clear light of day, and only when this is done will genuine, pure Homœopathy either fall away into deserved oblivion or, finally victorious, unite under her banner the whole medical world.

